# Characterizing healthcare worker attitudes toward the bivalent COVID-19 booster

**DOI:** 10.1017/ash.2023.300

**Published:** 2023-09-29

**Authors:** Kathryn Willebrand, Jacqueline Fredrick, Lauren Pischel, Kavin Patel, Scott Roberts, Thomas Murray, Richard Martinello

## Abstract

**Background:** Recent evidence has shown that the updated COVID-19 bivalent booster is effective in preventing COVID-19 compared with no previous vaccination and prior monovalent vaccination. Despite its effectiveness, uptake has been poor, and a minority of eligible recipients have received the booster. Understanding healthcare worker (HCW) attitudes for and against voluntary uptake of the bivalent booster dose against COVID-19 can help guide communication strategy to maximize uptake. In this survey study, we investigated attitudes toward updated and/or bivalent booster uptake in a behavioral health hospital shortly after a COVID-19 outbreak. **Methods:** A survey tool was developed and sent to all HCWs at the Yale New Haven Psychiatric Hospital in December 2022. The survey queried demographic data, job category, history of COVID-19, prior COVID-19 vaccinations, perception of COVID-19 exposure, and updated and/or bivalent booster doses. The survey was administered several weeks after a COVID-19 outbreak on multiple inpatient behavioral health units. Receipt of the COVID-19 primary vaccination series and the first booster dose were mandated for HCWs; however, receipt of the bivalent booster was voluntary. **Results:** The survey was sent to 664 HCWs with primary assignments in behavioral health settings. In total, 182 (27.4%) provided complete responses to the survey and are included in these data. Moreover, 91 HCWs (50.0%) reported previously having COVID-19 at least once. Overall, 100 HCWs (55.0%) received the bivalent booster. The most identified reasons for receiving the bivalent booster were wanting to protect family and friends (n = 113), importance of staying healthy (n = 112), and protecting colleagues and patients (n = 103). The most identified reasons for not wanting to receive the bivalent booster dose were not thinking it provides additional protection (n = 33), “too many” shots already received (n = 31), and concern about side effects (n = 30). **Discussion:** Bivalent booster dose uptake in HCWs on behavioral health units shortly after a COVID-19 outbreak was greater than the general population. HCWs reported varying reasons for and against receipt of the bivalent booster dose, with the most common being protection of family and friends and perceptions of no additional protection, respectively. A limitation of this study was voluntary response bias, in which results are biased toward individuals more likely to receive a bivalent booster vaccine. It is unclear whether reasons for declining the vaccine are representative of HCWs who did not complete the survey. Assessing attitudes for the bivalent booster dose can assist in guiding communication and outreach strategies to increase vaccine uptake by HCWs.

**Figure:**
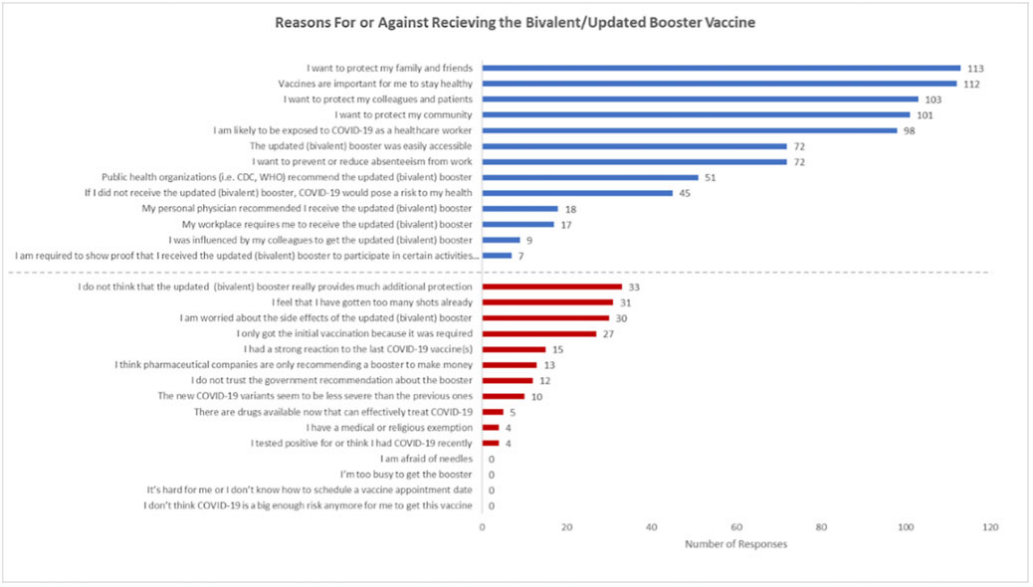

**Disclosures:** None

